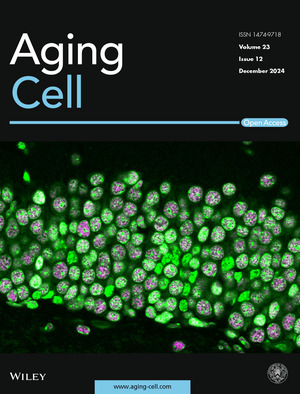# Additional Cover

**DOI:** 10.1111/acel.14456

**Published:** 2024-12-11

**Authors:** Ken‐ichi Takayama, Takashi Suzuki, Kaoru Sato, Yuko Saito, Satoshi Inoue

## Abstract

Cover legend: The cover image is based on the article *Cooperative nuclear action of RNA‐binding proteins PSF and G3BP2 to sustain neuronal cell viability is decreased in aging and dementia* by Satoshi Inoue et al., https://doi.org/10.1111/acel.14316.